# Anti-Hair Loss Potential of Perilla Seed Extracts: In Vitro Molecular Insights from Supercritical Fluid Extraction

**DOI:** 10.3390/foods14152583

**Published:** 2025-07-23

**Authors:** Anurak Muangsanguan, Warintorn Ruksiriwanich, Pipat Tangjaidee, Korawan Sringarm, Chaiwat Arjin, Pornchai Rachtanapun, Sarana Rose Sommano, Korawit Chaisu, Apinya Satsook, Juan Manuel Castagnini

**Affiliations:** 1 Department of Pharmaceutical Sciences, Faculty of Pharmacy, Chiang Mai University, Chiang Mai 50200, Thailand; anurak_m@cmu.ac.th; 2 Cluster of Valorization and Bio-Green Transformation for Translation Research Innovation of Raw Materials and Products, Chiang Mai University, Chiang Mai 50200, Thailand; korawan.s@cmu.ac.th (K.S.);; 3 Cluster of Agro Bio-Circular-Green Industry (Agro BCG), Chiang Mai University, Chiang Mai 50200, Thailand; pipat.t@cmu.ac.th (P.T.); pornchai.r@cmu.ac.th (P.R.); 4 Faculty of Agro-Industry, Chiang Mai University, Chiang Mai 50100, Thailand; korawit.chaisu@cmu.ac.th; 5 Department of Animal and Aquatic Sciences, Faculty of Agriculture, Chiang Mai University, Chiang Mai 50200, Thailand; chaiwat.arjin@cmu.ac.th; 6 Department of Plant and Soil Sciences, Faculty of Agriculture, Chiang Mai University, Chiang Mai 50200, Thailand; 7 Office of Research Administration, Chiang Mai University, Chiang Mai 50200, Thailand; apinya.satsook@cmu.ac.th; 8 Research Group in Innovative Technologies for Sustainable Food (ALISOST), Department of Preventive Medicine and Public Health, Food Science, Toxicology and Forensic Medicine, Faculty of Pharmacy, Universitat de València, Avenida Vicent Andrés Estellés s/n, 46100 Burjassot, Spain; juan.castagnini@uv.es

**Keywords:** 5α-reductase inhibition, *Perilla frutescens* (L.) Britt., androgenetic alopecia, maceration, screw extraction, transforming growth factor-beta

## Abstract

Perilla seed has long been recognized in traditional diets for its health-promoting properties, but its potential role in hair loss prevention remains underexplored. This study compared three extraction methods—maceration (MAC), screw pressing (SC), and supercritical fluid extraction (SFE)—to determine their efficiency in recovering bioactive compounds and their effects on androgenetic alopecia (AGA)-related pathways. The SFE extract contained the highest levels of polyunsaturated fatty acids and tocopherols, while MAC uniquely recovered a broader range of polyphenols. Among all extracts, SFE-derived perilla seed extract showed the most consistent biological effects, promoting proliferation of human hair follicle dermal papilla cells (HFDPCs) by 139.4 ± 1.1% at 72 h (*p* < 0.05). It also reduced TBARS and nitrite levels in HFDPCs to 66.75 ± 0.62% of control and 0.87 ± 0.01 μM, respectively, indicating strong antioxidant and anti-inflammatory effects. Importantly, the SFE extract significantly downregulated *SRD5A1-3* and *TGF-β1* expression—key genes involved in androgen-mediated hair follicle regression—outperforming finasteride, dutasteride, and minoxidil in vitro by approximately 1.10-fold, 1.25-fold, and 1.50-fold, respectively (*p* < 0.05). These findings suggest that perilla seed extract obtained via supercritical fluid extraction may offer potential as a natural candidate to prevent hair loss through multiple biological mechanisms. These in vitro results support its further investigation for potential application in functional food or nutraceutical development targeting scalp and hair health.

## 1. Introduction

Pattern hair loss, or androgenetic alopecia (AGA), is a common condition characterized by the gradual shrinking of hair follicles, ultimately leading to hair thinning and baldness. The hair follicle undergoes a cyclical process comprising the following four stages: anagen (growth), catagen (regression), telogen (rest), and exogen (shedding). Disruption of this cycle, particularly the premature transition from the anagen to the catagen phase, is crucial in the initiation of AGA. One of the key contributors to this early transition is dihydrotestosterone (DHT), a potent androgen converted from testosterone (TT) by the action of 5*α*-reductase (SRD5A) enzymes. Elevated DHT levels in androgen-sensitive areas of the scalp accelerate the transition from the anagen phase to the catagen phase, leading to shortened hair cycles and follicle miniaturization [1,2].

In addition to androgen activity, the transforming growth factor-beta (TGF-β) family, particularly TGF-β1, plays a critical role in this process. Under the influence of DHT, human hair follicle dermal papilla cells (HFDPCs) upregulate *TGF-β1* expression, which suppresses epithelial cell proliferation and induces apoptosis. These effects promote the transition from the anagen to the catagen phase, contributing to hair follicle regression. Thus, TGF-β1 acts as a downstream effector of DHT, promoting hair loss through both growth-inhibitory and pro-apoptotic effects [3].

Current pharmaceutical interventions, including oral finasteride (a selective SRD5A2 inhibitor) and topical minoxidil, are commonly employed in the treatment of AGA. However, these treatments are frequently accompanied by undesirable side effects, such as decreased libido, hormonal imbalances, and skin irritation [1]. Furthermore, recent studies and market analyses have revealed a significant rise in consumer preference for natural-based products, largely driven by increasing concerns over long-term safety, sustainability, and the desire for fewer side effects. For example, a 2024 global study reported that over 44% of consumers prefer plant-based cosmetics and supplements to address cosmetic concerns, including hair loss [4]. Plant-based remedies are gaining attention as promising options for supporting hair growth and reducing hair loss due to their potential to deliver effective results with fewer safety concerns.

*Perilla frutescens* (L.) Britt., commonly known as Zisu, is a member of the Lamiaceae family and is extensively cultivated in East Asia, particularly in China, Korea, Japan, and Thailand [5]. In Thailand, perilla is primarily grown in the northern regions. *P. frutescens* has also been traditionally used in East Asia not only as a food ingredient but also for its medicinal properties, particularly its anti-inflammatory and antioxidant effects, which have been demonstrated in in vitro studies [6]. Previous research has shown that perilla seeds are a rich source of oil, comprising approximately 30–45% of the seed’s weight. The oil is abundant in several bioactive constitutes, including polyphenols and tocopherols, which are well-recognized for their antioxidant properties [7]. Additionally, perilla seed oil contains considerable quantities of fatty acids, including saturated fatty acids (SFAs; e.g., palmitic acid), monounsaturated fatty acids (MUFAs; e.g., oleic acid), and polyunsaturated fatty acids (PUFAs; e.g., linoleic acid) [7]. These components have demonstrated potential in preventing hair loss through antioxidant, anti-inflammatory effects, and by downregulating alopecia-related genes, including *SRD5A* and *TGF-β* [8,9]. Specifically, polyphenols such as ferulic acid, phytic acid, and gallic acid, as well as *α*-, *β*-, *γ*-, and *δ*-tocopherols and various fatty acids, have been shown to suppress 5α-reductase activity, thereby lowering DHT levels and mitigating androgen-induced follicle miniaturization. [8,9].

To optimize the recovery of bioactive compounds from perilla seeds, the following three extraction methods were evaluated: maceration (MAC), supercritical fluid extraction (SFE), and screw pressing (SC). MAC is a traditional solvent-based technique in which plant materials are soaked in a solvent to facilitate the diffusion of bioactive compounds. This method is effective for extracting both polar and non-polar compounds; however, it is time-consuming and requires large volumes of solvent [10]. SC is a mechanical method that applies pressure to extract oils and bioactive compounds from plant materials, offering high oil yields without degrading heat-sensitive components [11]. Additionally, SFE is an emerging green technology that utilizes supercritical CO_2_ as the solvent. It operates at relatively low temperatures, thereby minimizing oxidative degradation of heat-sensitive compounds. Moreover, SFE provides high-purity extracts, making it suitable for applications in pharmaceuticals and cosmetics [10].

This study aims to investigate the effects of different extraction methods (MAC, SC, and SFE) on maximizing the yield of bioactive compounds from perilla seeds. Additionally, the biological activities of the extracts were evaluated through in vitro testing, focusing on their antioxidant and anti-inflammatory properties. Although *P. frutescens* seeds are known to contain several bioactive compounds, their potential application in treating AGA has not yet been explored. Moreover, the biological activity of perilla seed extracts—particularly in relation to hair loss pathways—has not been evaluated using different extraction approaches. Importantly, this study presents the first investigation into the potential of perilla seed extracts to interfere with key drivers of hair loss by targeting critical pathways, including *SRD5A* isoforms (types 1, 2, and 3) and *TGF-β*, both of which are implicated in hair follicle shrinkage.

## 2. Materials and Methods

### 2.1. Chemicals and Reagents

The following chemicals and reagents were utilized in this study: 2,2-azino-bis (3-ethylbenzothiazoline-6-sulfonic acid) (ABTS), 2,2-diphenyl-l-picrylhydrazyl (DPPH), diclofenac sodium, dimethyl sulfoxide (DMSO), epigallocatechin gallate (EGCG), gallic acid, L-ascorbic acid, lipopolysaccharide (LPS) solution, sulforhodamine B (SRB), and Trolox (6-hydroxy-2,5,7,8-tetramethylchroman-2-carboxylic acid) were procured from Sigma-Aldrich (St. Louis, MO, USA). Aluminum chloride hexahydrate, Folin–Ciocalteu reagent, hydrogen peroxide, Triton X-100, and trichloroacetic acid (TCA) were obtained from Merck (Darmstadt, Germany). Tolbutamide (TBT) was sourced from MedChemExpress (Monmouth Junction, NJ, USA). Thiobarbituric acid (TBA) was acquired from VWR Chemicals (BDH Chem. Ltd., Poole, UK). Finasteride, dutasteride, and minoxidil were purchased from Wuhan W&Z Biotech (Wuhan, China).

Cell culture reagents included Dulbecco’s Modified Eagle Medium (DMEM) and Roswell Park Memorial Institute 1640 Medium (RPMI-1640), along with fetal bovine serum (FBS), penicillin–streptomycin (100×), and 1× phosphate-buffered saline (PBS) solution, all obtained from Gibco Life Technologies (Thermo Fisher Scientific, Waltham, MA, USA). The primary fibroblast growth medium kit (ready to use) (catalog no. PriMed-iCELL-003) was purchased from iCell Bioscience Inc. (Shanghai, China). The Griess reaction colorimetric kit was obtained from Invitrogen (Thermo Fisher Scientific, Inc., Eugene, OR, USA). Unless otherwise specified, the remaining chemicals employed in this research were of analytical grade.

The software used in this study included LabSolutions (version 1.86; Shimadzu, Kyoto, Japan) and Image Lab™ (version 5.1; Bio-Rad Laboratories, Hercules, CA, USA).

### 2.2. Plant Extract Preparation

Perilla seeds used in this study were sourced from a local market in Chiang Mai during November to December 2024. A voucher specimen of the plant (PNPRDU67001) was collected by *P. Tangjaidee*, and it is preserved at the Pharmaceutical and Natural Products Research and Development Unit (PNPRDU), Chiang Mai University. The seeds were initially washed with tap water and subsequently dried in an oven at 60 °C for 6 h until the moisture content was reduced below to 10% (*w*/*v*).

For screw press (SC) extraction [11], dried seeds were subjected to mechanical extraction using an LT-RG312 screw press machine from Wenzhou, China, operated under the following conditions: die diameter of 2.5 cm, pressing temperature of 120 °C, extraction duration of 10 min, and a feed rate of 0.05 kg/h. The dried seeds were not ground prior to extraction due to the high oil content in perilla seeds, as grinding could lead to excessive oil release, resulting in waste. After the screw press extraction, the residual seed cake was further extracted using the maceration (MAC) method. For MAC [2], seed cakes (20 g) were soaked in 95% (*v*/*v*) ethanol (100 mL) at room temperature for 48 h with intermittent stirring.

In parallel, supercritical fluid extraction (SFE) was carried out using an SFE system (Separations, Pittsburgh, PA, USA) [12]. Briefly, 20 g of dried seeds were blended with 9.00 mL of ethanol (used as a co-solvent) and loaded into the SFE vessel. The extraction was performed under standardized conditions: extraction time of 10 min, temperature of 40 °C, and pressure of 416 bar. Following extraction, all samples from each method were filtered through Whatman No. 1 filter paper and subsequently concentrated using a rotary evaporator at 40–50 °C until complete removal of the solvent.

### 2.3. Phytochemical Evaluation

#### 2.3.1. Evaluation of Total Phenolic Content

The total phenolic content of seed extracts was quantified using the Folin–Ciocalteu colorimetric method with slight modifications from previously reported protocols [2]. Briefly, the seed extracts were reacted with Folin–Ciocalteu reagent, followed by the addition of saturated sodium bicarbonate. After incubation at 25 °C for 2 h, absorbance was measured at 765 nm using a UV-Vis spectrophotometer (EZ Read 400 Flexi, Biochrom, UK). Gallic acid was employed to generate the standard calibration curve at concentrations of 0–200 μg/mL. The calibration curve exhibited a linear relationship, with the regression equation *y* = 8.2493x + 0.0398 and a correlation coefficient (R^2^) of 0.9981. Results were expressed as milligrams of gallic acid equivalents per gram of extract (mg GAE/g extract).

#### 2.3.2. Evaluation of Polyphenol Composition

The polyphenol profile was analyzed as previously described [8], with slight adjustments. Briefly, perilla seed extracts were diluted with 50% (*v*/*v*) ethanol and filtered through a 0.45 μm syringe filter. Shimadzu high-performance liquid chromatography (HPLC) was performed coupled with a photo-diode array detector (CTO-20AC, Shimadzu, Kyoto, Japan) at 280 nm. Separation was conducted on a Restek Ultra C18 column (250 × 4.6 mm, 5 μm, Restek, Bellefonte, PA, USA) with reverse-phase chromatography. The mobile phase consisted of solution A (5% formic acid, and 95% water) and solution B (85% acetonitrile, 10% water, and 5% formic acid). The linear gradient program was as follows: 20% B (0–4 min), 20–75% B (4–12 min), hold at 75% B (12–14 min), decreased to 30% B (14–17 min), and to 5% B (17–18 min). The flow rate was 1 mL/min, with a 10 μL injection volume, and the column temperature was maintained at 40 °C.

#### 2.3.3. Evaluation of Tocopherol Composition

Tocopherol content in the seed extracts was analyzed using HPLC, following a previously described method [8] with minor adjustments. The analysis employed a Shimadzu HPLC system (Kyoto, Japan) equipped with a fluorescence detector (RF-20A) and a reversed-phase Restek Ultra C18 column. The mobile phase consisted of two solvent mixtures: solvent A (acetonitrile, methanol, and isopropanol in a ratio of 50:40:10) and solvent B (30:65:5). A gradient elution program was applied as follows: the run began with 85% solvent A, maintained for first 15 min, then reduced to 10% over 2 min, elevated to 50% within 5 min, and finished by returning to 85% over the last 3 min. The total runtime was approximately 26 min, with a constant flow rate at 1.0 mL/min. Fluorescence detection used excitation at 290 nm and emission at 330 nm. Identification of tocopherol homologues was based on retention time comparisons with standard tocopherols. Each isoform was quantified using specific calibration curves, and the concentrations were reported for each tocopherol isoform in the seed extracts.

#### 2.3.4. Evaluation of Antioxidant Activities

##### ABTS Radical Scavenging Assay

The ability of the seed extracts to scavenge ABTS radicals was following an earlier method [13]. ABTS radicals were generated by incubating 7.0 mM ABTS and 2.45 mM potassium persulfate in the dark at room temperature for 12–16 h. Prior to use, the solution was diluted with 95% (*v*/*v*) ethanol to an absorbance of 0.70 ± 0.02 at 734 nm. For the assay, each extract sample was mixed with the ABTS working solution, incubated at 25 °C for 30 min in the dark, and absorbance was recorded at 734 nm. Trolox was used as the reference antioxidant, and ethanol as the blank. The percentage of radical scavenging activity was calculated using the following equation:

(1) $$\mathrm{ABTS}\;\mathrm{radical}\;\mathrm{scavenging}\;\mathrm{activity}\;\left(\%\right)=\frac{\left(A_{\;\mathrm{control}}-{A\;}_{\mathrm{sample}}\right)}{{A\;}_{\mathrm{control}}}\;\times \;100\;$$

where ${A\;}_{\mathrm{control}}$ is the absorbance of the ABTS solution with ethanol and ${A\;}_{\mathrm{sample}}$ is the absorbance of the ABTS solution with the extract.

##### DPPH Radical Scavenging Assay

To evaluate the antioxidant potential of the extracts, their ability to scavenge free radicals were examined using the DPPH assay [13]. A fresh 0.1 mM DPPH solution in 95% (*v*/*v*) ethanol was prepared. Extracts were mixed with DPPH solution and incubated in the dark at 25 °C for 30 min. Absorbance was then recorded at 515 nm. Trolox was used as the reference antioxidant, and ethanol as the blank. The DPPH scavenging activity was calculated using the following formula:

(2) $$\mathrm{DPPH}\;\mathrm{radical}\;\mathrm{scavenging}\;\mathrm{activity}\;\left(\%\right)=\frac{\left({A\;}_{\mathrm{control}}-{A\;}_{\mathrm{sample}}\right)}{A_{\;\mathrm{control}}}\;\times \;100$$

where ${A\;}_{\mathrm{control}}$ is the absorbance of the DPPH solution with ethanol and ${A\;}_{\mathrm{sample}}$ is the absorbance of the DPPH solution with the extract.

##### Ferric Reducing Antioxidant Power (FRAP) Assay

The FRAP assay was employed to determine the antioxidant reducing capacity of the seed extracts, following a previously described protocol with slight modifications [14]. The FRAP reagent was freshly prepared by mixing 10 mM TPTZ (in 40 mM HCl), 20 mM FeCl_3_, and 0.3 M acetate buffer (pH 3.6) at a ratio of 1:1:10. In a 96-well plate, each extract was combined with the FRAP reagent and incubated at 25 °C for 30 min in the dark. The absorbance was recorded at 595 nm. Ferrous sulfate was used as the standard antioxidant, and ethanol as the blank. The results were expressed as millimoles of ferrous ion equivalents per gram of extract (mM Fe^2+^/g extract).

##### Lipid Peroxidation Inhibition Using Ferric Thiocyanate (FTC) Assay

The inhibition of lipid peroxidation by seed extracts was evaluated using the FTC assay, with minor modifications from a standard method [15]. In a 96-well plate, each extract sample was mixed with 1.3% (*v*/*v*) linoleic acid in PBS and 20 mM AAPH in PBS, followed by incubation at 37 °C for 4 h. After incubation, 10% (*w*/*v*) ammonium thiocyanate and 20 mM FeCl_2_ solutions were added to the mixture and incubated for an additional 3 min. The absorbance was measured at 500 nm. Trolox was used as the reference antioxidant, and ethanol as the blank. The results were expressed as milligrams of Trolox equivalent antioxidant capacity per gram of extract (mg TEAC/g extract).

#### 2.3.5. Evaluation of Fatty Acid Composition

Fatty acid profiles were determined by gas chromatography using fatty acid methyl esters (FAMEs), prepared following a standard protocol [16] with slight modifications. Gas chromatography analysis was conducted using a Shimadzu GC-2030 system (Kyoto, Japan) installed with a flame ionization detector (FID) and RT-2560 capillary column (100 m × 0.25 mm × 0.25 μm, Restek, USA). Helium served as the carrier gas, with injector and detector temperatures maintained at 250 °C. The oven temperature was programmed to start at 100 °C and increased steadily by 3 °C per min until 240 °C, where it was held for an additional 20 min. Using these settings, FAME samples were subjected to injection. Chromatograms were processed using LabSolutions software (version 1.86; Shimadzu, Kyoto, Japan). Identification of fatty acid components were performed by matching their retention times with those of reference standards. The results were presented as the percentage of each fatty acid relative to the total fatty acid content of the sample.

### 2.4. Measurement of Cell Viability and Proliferation

This study utilized the following cell lines: hTERT-immortalized human dermal fibroblasts (hTERT fibroblasts), human keratinocytes (HaCaT), murine macrophages (RAW 264.7), and human prostate cancer cells (DU-145), all sourced from the American Type Culture Collection (ATCC, Rockville, MD, USA). HFDPCs were obtained through iCell Bioscience Inc. (Shanghai, China). hTERT fibroblasts, HaCaT keratinocytes, and RAW 264.7 cells were cultured in DMEM, while DU-145 cells were maintained in RPMI-1640 medium. Both media were supplemented with 10% FBS and 1% penicillin–streptomycin (100×). HFDPCs were grown in a primary fibroblast-specific medium provided by the supplier.

To evaluate the cytotoxicity and proliferative effects of the seed extracts and standard reference compounds (diclofenac sodium, dutasteride, finasteride, and minoxidil), SRB assays were performed. Cells were seeded into 96-well plates at a density of 1 × 10^5^ cells/mL and allowed to attach for 24 h in a 5% CO_2_ incubator at 37 °C. Treatments were then applied at concentrations ranging from 0.063 to 2.000 mg/mL and incubated for an additional 24 h. After treatment, cells were fixed using cold 50% (*w*/*v*) TCA for 1 h, stained with 0.04% (*w*/*v*) SRB solution for 30 min, and washed with 10 mM Tris base to remove excess dye. Absorbance readings were taken at 515 nm. Cell viability was calculated using Equation (3), and only concentrations maintaining ≥80% viability were considered safe for further experiments [8]. Equation (3) is as follows:

(3) $$\mathrm{Cell}\;\mathrm{viability}\;\left(\%\right)=\frac{\left(A_{\;\mathrm{sample}}-{A\;}_{\mathrm{blank}}\right)}{\left(A_{\;\mathrm{control}}-{A\;}_{\mathrm{blank}}\right)}\;\times \;100$$

where ${A\;}_{\mathrm{sample}}$ is the absorbance of cells treated with the test sample; ${A\;}_{\mathrm{control}}$ is the absorbance of untreated cells (vehicle control); and ${A\;}_{\mathrm{blank}}$ is the absorbance of wells containing serum-free medium and reagents without cells (background signal).

### 2.5. Anti-Inflammatory Activity Assay

To assess nitric oxide (NO) production, nitrite accumulation in the culture medium was determined using a commercially available Griess reagent-based colorimetric assay. RAW 264.7 cells and HFDPCs were seeded at a density of 1 × 10^5^ cells/mL and incubated for 24 h. The cells were subsequently pre-treated with either the seed extracts, diclofenac sodium (positive control), or serum-free medium (blank) for 2 h. Then, 1 μg/mL LPS was added to stimulate an inflammatory response, and the cells were incubated for an additional 24 h. Following treatment, the culture supernatants were harvested and reacted with the Griess reagent. Nitrite levels, representing NO production, were quantified using a standard curve generated with sodium nitrite solutions ranging from 0.01 to 50 μM [2].

### 2.6. Thiobarbituric Acid-Reactive Substances (TBARS) Assay

The antioxidant capacity of the seed extracts was evaluated through the TBARS assay, utilizing L-ascorbic acid as the reference standard and serum-free medium as the untreated control. HFDPCs were plated in 6-well plates (1 × 10^5^ cells/mL) and cultured for 24 h. The cells were then treated with either the seed extract or the reference standard (0.125 mg/mL) for 24 h. Oxidative stress was induced by exposing the cells to hydrogen peroxide (H_2_O_2_) for 2 h. After treatment, the cells were lysed and incubated with a reaction mixture containing 1% Triton X-100, 0.6% TBA, and 15% TCA. The mixture was heated at 100 °C for 10 min before being quickly cooled to −80 °C for 10 min to stop the reaction. Absorbance was measured at 532 nm to quantify malondialdehyde (MDA) levels, a key marker of lipid peroxidation, relative to the untreated control [2].

### 2.7. Semi-Quantitative Reverse Transcription and Polymerase Chain Reaction Analysis

The mRNA expression of hair loss-associated genes—specifically those involved in the androgen pathway (*SRD5A1*, *SRD5A2*, and *SRD5A3*) and the TGF-β signaling pathway (*TGF-β1*), which plays a key role in hair follicle cycling and fibrosis—was evaluated in DU-145s and HFDPCs using semi-quantitative RT-PCR [2]. Cells were seeded in 6-well plates at a density of 1 × 10^5^ cells/mL and cultured for 24 h. After incubation, the cells were treated with seed extracts or standard reference compounds, including dutasteride, finasteride, and minoxidil, at a final concentration of 0.125 mg/mL for 24 h.

Total RNA was isolated using the E.Z.N.A.^®^ Total RNA Kit I (Omega BioTek, Norcross, GA, USA). RNA purity and concentration were determined using a NanoDrop spectrophotometry (Thermo Fisher Scientific, Waltham, MA, USA). Reverse transcription and PCR amplification were performed with the MyTaq™ One-Step RT-PCR Kit (Bioline, Memphis, TN, USA). Primer sequences for *SRD5A1-3*, *TGF-β1*, and *GAPDH* (used as the internal reference gene) are listed in Table 1. PCR products were resolved by agarose gel electrophoresis, and band intensities were captured using the Gel Doc™ EZ Imaging System (Bio-Rad Laboratories, Hercules, CA, USA). Semi-quantitative analysis was conducted using Image Lab™ software (Version 5.1), and gene expression levels were reported as fold changes relative to vehicle-treated control cells.

## 3. Results and Discussion

### 3.1. Extraction Yield, Bioactive Constituents, and Scavenging Potential of Perilla Seed Extracts

Table 2 summarized the extraction yields (expressed as percentages) obtained from perilla seeds using the following three different extraction methods: maceration (MAC-PS), supercritical fluid extraction (SFE-PS), and screw extraction (SC-PS). The data revealed that extraction efficiency significantly depended on the method employed. Among the tested approaches, MAC-PS yielded the highest extraction efficiency (42.34 ± 0.11%), followed by SFE-PS with a yield of 33.93 ± 0.28%. The lowest yield was observed with SC-PS, which produced 22.46 ± 0.20%. These findings aligned with previous studies by Vieitez et al. [17] and Melo et al. [18], who documented comparable yields between maceration and supercritical fluid extraction for various plant materials, including rosemary, boldo, pitanga, and yerba mate. While screw extraction offered advantages including operational simplicity and a solvent-free process, its comparatively lower yield was likely attributable to reliance on pressure alone, which may have been insufficient to disrupt the cellular structure of plant material and release bioactive compounds. Similar limitations have been noted in oilseed and herbal processing studies, where solvent-based and supercritical fluid techniques consistently achieved higher extraction efficiencies and improved recovery of bioactive constituents [19].

The total phenolic content of perilla seed extracts is summarized in Table 2. Among the three extraction methods, MAC-PS demonstrated the highest total phenolic content at 23.07 ± 0.32 mg GAE/g extract, followed by SFE-PS at 8.22 ± 0.73 mg GAE/g extract. SC-PS showed the lowest value, at 1.17 ± 0.39 mg GAE/g extract. These results suggested that both solvent polarity and extraction mechanism critically influenced phenolic compound recovery. The greater efficiency of MAC-PS and SFE-PS in extracting phenolics was attributed to the use of ethanol as a solvent, which enhances the solubility and diffusion of phenolic compounds [20]. This observation aligned with previous findings that reported higher phenolic content in ethanolic macerates of yerba mate compared to samples extracted via supercritical fluid extraction [17]. In contrast, the markedly lower phenolic content obtained from SC-PS likely reflected the absence of solvent and reliance solely on pressure, which may have been insufficient to rapture cell walls of plant and release phenolic compounds contained inside [21].

Furthermore, MAC-PS was the only extraction method that allowed the detection of a broader variety of polyphenolic compounds, including gallic acid, epicatechin, caffeic acid, epicatechin gallate, naringin, rosmarinic acid, *o*-coumaric acid, *p*-coumaric acid, and quercetin, as shown in the HPLC chromatograms (Supplementary Figure S1). In comparison, while SFE-PS also extracted caffeic acid, its concentration (12.80 ± 0.00 μg/g extract) was lower than that found in the MAC-PS extract (17.54 ± 0.28 μg/g extract). These findings were consistent with the total phenolic content results and suggested that caffeic acid was a major phenolic constituent in perilla seed [22]. Previous studies have also shown that the polarity of co-solvents, particularly ethanol, plays a significant role in the efficiency of polyphenol extraction [23]. Notably, semi-polar polyphenols such as caffeic acid and rosmarinic acid have been reported to exert anti-hair loss effects by downregulating expression of genes involved in the androgen signaling [24].

Tocopherols, a group of vitamin E compounds, are potent lipophilic antioxidants naturally found in various plant oils [25]. The extraction method plays a crucial role in preserving the nutritional and functional properties of plant-derived extracts, particularly those intended for pharmaceutical or nutraceutical applications [26]. In this study, the tocopherol profile of perilla seed extracts was significantly influenced by the extraction method. Tocopherol compounds were not detected in MAC-PS, whereas both SFE-PS and SC-PS contained notable levels of *α*-, *β*- and *γ*-, and *δ*-tocopherol, with SC-PS exhibiting the highest concentrations. Although SFE is commonly employed for extracting lipophilic antioxidants such as tocopherols and oryzanols, SC-PS demonstrated superior efficiency in tocopherols extraction. This may be attributed to its solvent-free mechanical oil extraction process, which effectively recovers non-polar compounds present in the oil phase. Moreover, SC generates only moderate heat through mechanical compression, thereby minimizing thermal and oxidative degradation of thermolabile antioxidants in plant extracts [27]. These characteristics make SC a green and industry-friendly alternative for high-quality oil extraction. Among the detected isoforms, *β*- and *γ*-tocopherol were predominant, with concentrations reaching 513.16 ± 3.77 μg/g in SC-PS. This finding suggested that SC not only preserved antioxidant compounds but also effectively concentrated key antioxidant components. The HPLC chromatograms of tocopherol compounds are shown in the Supplementary Materials (Figure S2).

Tocopherols have been well-known for their role in mitigating oxidative damage [28]. In this study, the tocopherol content was consistent with the antioxidant profiles observed. In both ABTS and DPPH assays, MAC-PS exhibited the highest radical scavenging activity (85.72 ± 0.96% and 75.70 ± 2.71%, respectively), which was likely due to its elevated total phenolic content (23.07 ± 0.32 mg GAE/g extract) and rich polyphenolic composition.

However, in assays that better reflected lipophilic antioxidant capacity, such as FRAP and FTC, both SFE-PS and SC-PS outperformed MAC-PS. Specifically, SFE-PS showed the highest FRAP value at 121.21 ± 2.72 mM Fe^2+^/g extract, followed by SC-PS (92.38 ± 1.14 mM Fe^2+^/g extract) and MAC-PS (68.32 ± 2.11 mM Fe^2+^/g extract). This result may be attributed to the combined presence of tocopherols and relatively high levels of phenolic compounds, which functioned as effective reducing agents in the FRAP assay [29]. In contrast, SC-PS exhibited the highest FTC value at 19.53 ± 1.04 mg TEAC/g extract, followed by SFE-PS (17.38 ± 1.02 mg TEAC/g extract) and MAC-PS (9.85 ± 1.36 mg TEAC/g extract). This finding emphasized the strong electron-donating ability and lipid peroxidation-inhibitory effects associated with the tocopherol-rich composition of SC-PS [30].

Despite SC-PS exhibiting the highest total tocopherol content, SFE-PS showed superior antioxidant activity in ABTS, DPPH, and FRAP assays. This variation may be attributed to the unique presence of caffeic acid in SFE-PS, which was not detected in SC-PS. Caffeic acid is a highly potent hydrophilic phenolic compound with strong radical scavenging and metal-reducing capacity [31]. The presence of caffeic acid likely contributed synergistically to the superior antioxidant activity of SFE-PS observed in ABTS, DPPH, and FRAP assays. In contrast, tocopherols are more effective in inhibiting lipid peroxidation, which explains why SC-PS exhibited the highest activity in the FTC assay. These results highlight that antioxidant potential depends not only on the quantity of tocopherols but also on the qualitative composition and solubility of antioxidant compounds and the type of assay employed [32].

AGA pathogenesis involves a substantial role of oxidative stress and other types of hair loss by inducing premature senescence of HFDPCs and disrupting key signaling pathways essential for hair regeneration. The tocopherol compounds identified in this study, particularly *β*- and *γ*-tocopherol, possess both scavenging and inflammation suppression effects that may help reduce ROS-induced damage in hair follicles [33]. This protective effect could contribute to the stabilization of cellular redox balance, thereby supporting the prolongation of the anagen phase of the hair cycle. Moreover, lipid peroxidation in scalp hair follicles has been associated with follicle miniaturization and hair shaft thinning [34]. The strong FTC performance observed in SC-PS and SFE-PS suggested their potential to protect hair follicles from oxidative degradation, which may ultimately prevent hair loss. These results provide a strong basis for subsequent in vitro studies to evaluate the ability of SC-PS and SFE-PS in promoting hair follicle regeneration in the future.

The fatty acid profiles of perilla seed extracts obtained using three different extraction methods—MAC-PS, SFE-PS, and SC-PS—were presented in Table 3. The GC-FID chromatograms of fatty acid profiles from MAC-PS, SFE-PS, and SC-PS extracts are presented in Supplementary Figure S3. Notably, maceration with ethanol as the solvent (MAC-PS) demonstrated no detectable levels of fatty acids. This result suggested that ethanol-based maceration was ineffective for extracting lipid-based compounds from perilla seeds. This finding corresponded to previous research that highlighted the limitations of solvent-based maceration in recovering non-polar constituents [35].

In contrast, both SFE-PS and SC-PS extracts contained significant levels of saturated fatty acids (SFAs), monounsaturated fatty acids (MUFAs), and polyunsaturated fatty acids (PUFAs), with comparable overall compositions between the two methods (*p* > 0.05). Among the SFAs, palmitic acid was the predominant fatty acid, found at 6.83% in SFE-PS and 6.88% in SC-PS, followed by stearic acid and butyric acid. The MUFAs profile was dominated by oleic acid, accounting for approximately 11.75% in both extraction methods. The role of oleic acid in enhancing hair growth has been well-documented [36]. Furthermore, the main PUFA components were *α*-linolenic acid (59.43% in SFE-PS and 59.23% in SC-PS) and linoleic acid (17.45% in SFE-PS and 17.36% in SC-PS). Both fatty acids have been associated with reducing inflammation and oxidative stress within hair follicles, which could lengthen the anagen phase of the hair cycle [37]. Moreover, linoleic acid has been reported to promote the proliferation of HFDPCs, thereby promoting hair growth [36]. Notably, previous studies have shown that fatty acid-rich rice bran extracts, particularly those high in linoleic acid, can suppress the expression of *SRD5A*, a gene known to play a pivotal role in androgenetic alopecia [37].

### 3.2. Effect of Perilla Seed Extracts on Cell Viability and Proliferation

Cell viability and proliferation assays were conducted to assess the cytotoxic effects of perilla seed extracts with concentrations between 0.063 and 2.000 mg/mL on RAW 264.7, hTERT fibroblasts, HaCaT keratinocytes, DU-145 cells, and HFDPCs. In accordance with ISO 10993-5 standards, a viability greater than 80% is considered as non-toxic to cells [38]. After 24 h of treatment, concentrations ≥0.250 mg/mL induced cytotoxicity in RAW 264.7, HaCaT keratinocytes, and DU-145 cells, while concentrations ≥0.500 mg/mL were cytotoxic to hTERT fibroblasts and HFDPCs, relative to untreated controls. Detailed cell viability data for all concentrations and all cell types are provided in Supplementary Table S1. To ensure a safe and consistent concentration across all cell types, 0.125 mg/mL—maintaining viability above the 80% threshold—was selected for subsequent experiments.

hTERT fibroblasts and HaCaT keratinocytes were included in the cytotoxicity screening to broaden the assessment across relevant cell types. These cell types represent human dermal fibroblasts and epithelial cells, both of which are involved in hair follicle expansion. Although not used in downstream functional assays, their inclusion confirmed that the selected concentration was non-toxic to all relevant cell types. Both hTERT fibroblasts and HaCaT keratinocytes play important roles in hair follicle expansion and regeneration. However, the activity of these cells depends on signaling from HFDPCs, which activate the necessary pathways to promote hair follicle regeneration and growth [39].

**Figure 1:**
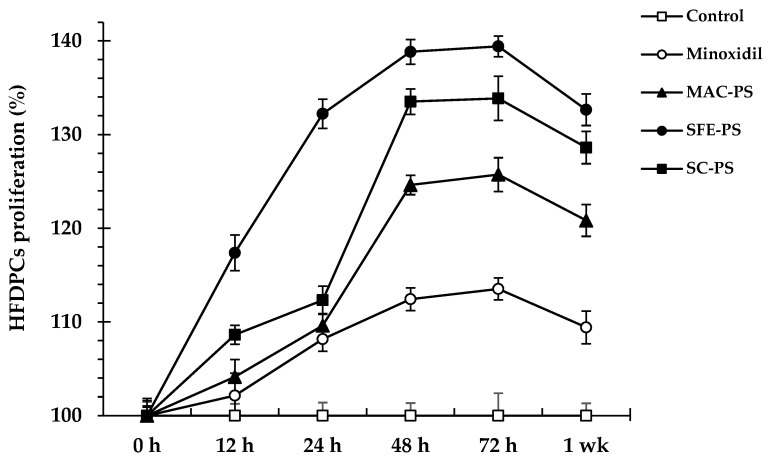
Proliferation of HFDPCs at 0, 12, 24, 48, 72 h, and 1 week upon exposure to perilla seed extracts or the standard treatment (minoxidil), at 0.031 mg/mL, relative to the untreated control. Data are reported as mean ± SD. Abbreviations: MAC-PS, macerated perilla seed extract; SFE-PS, supercritical fluid-extracted perilla seed extract; SC-PS, screw-pressed perilla seed extract; ND, not detected.

To assess the proliferative effects of perilla seed extracts on HFDPCs, cell proliferation was monitored at multiple time points (0, 12, 24, 48, 72 h, and 1 week) after exposure to 0.031 mg/mL of each extract. All extracts significantly enhanced HFDPC proliferation compared to both the untreated and minoxidil-treated controls (*p* < 0.05) across all time points (Figure 1). Notably, SFE-PS demonstrated the strongest proliferative effect, reaching 117.38 ± 1.90% at 12 h, peaking at 139.42 ± 1.10% at 72 h, and maintaining a high level of 132.65 ± 1.36% at 1 week. Minoxidil, a well-established hair growth agent, promotes HFDPC proliferation primarily by activating the ERK and AKT signaling pathways, which enhance cell survival and proliferation. These pathways protect HFDPCs from apoptosis and stimulate the mitogenic response necessary for follicular regeneration. Therefore, minoxidil served as a positive control in this study [40].

HFDPCs are essential regulators of hair follicle development and cycling. Hair follicle morphogenesis involves the following three key stages: induction, organogenesis, and cytodifferentiation. During the induction phase, HFDPCs release inductive signals that stimulate the proliferation and differentiation of overlying epithelial cells, initiating the formation of the placode and basal plate. In the organogenesis phase, the follicular structure extends into the dermis to form hair germs, while HFDPCs further stimulate keratinocyte proliferation, promoting the formation of dermal papillae. In the final cytodifferentiation stage, the hair follicle undergoes terminal maturation, resulting in the formation of the inner root sheath and hair shaft [2,41].

Among all extracts tested, SFE-PS exhibited the highest efficacy in stimulating HFDPC proliferation at all time points, outperforming both SC-PS and MAC-PS. This enhanced activity was likely attributable to its higher content of bioactive compounds, particularly tocopherols, polyphenols, and unsaturated fatty acids such as linoleic and oleic acids. These compounds are known to support cell proliferation and survival through antioxidant, anti-inflammatory, and mitogenic mechanisms, which in turn enhanced the signaling functions of HFDPCs in promoting hair growth [42].

### 3.3. Effect of Perilla Seed Extracts on Anti-Inflammatory Activities

The accumulation of nitric oxide (NO), a key mediator in inflammation, contributes to scalp inflammation and ultimately results in hair loss. Various factors, including oxidative stress, tissue damage, microbial infection, and elevated DHT, are implicated in promoting NO accumulation in hair follicles, leading to follicular inflammation. Under inflammatory conditions, perifollicular macrophages are activated in response to oxidative stress, DHT, or other pro-inflammatory stimuli. Once activated, these macrophages release significant amounts of NO, which further promotes inflammation within the hair follicle, ultimately contributing to hair loss [43,44].

**Figure 2:**
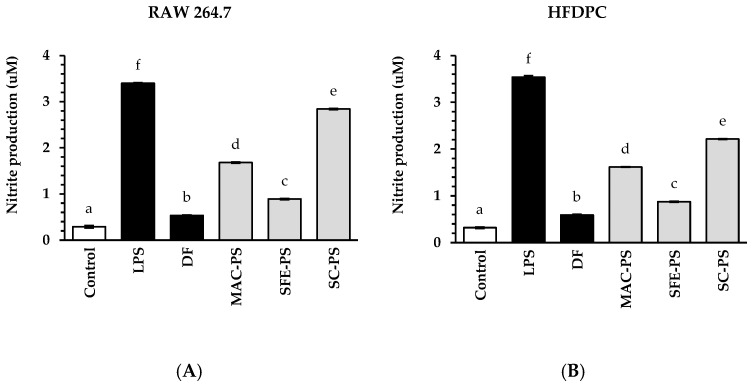
The inhibitory effect of perilla seed extracts on nitrite production is evaluated in lipopolysaccharide (LPS)-induced RAW 264.7 (**A**) cells and HFDPCs (**B**). Results are compared against a negative control (LPS-induced cells without pre-treatment), standard treatment (DF: diclofenac sodium), and untreated control at 0.125 mg/mL. Data are reported as mean ± SD for each sample. Statistical comparisons are applied through a one-way ANOVA followed by Tukey’s post hoc test. Distinct lowercase letters (a–f) represent statistically significant differences among groups in each assay (*p* < 0.05). Abbreviations: MAC-PS, macerated perilla seed extract; SFE-PS, supercritical fluid-extracted perilla seed extract; SC-PS, screw-pressed perilla seed extract.

In this study, perilla seed extracts and a standard anti-inflammatory drug (diclofenac sodium, DF) at 0.125 mg/mL were assessed for their ability to suppress NO production in RAW 264.7 macrophages and HFDPCs (Figure 2). The results showed that NO levels in the LPS-treated group were significantly greater than those in the untreated control (blank) in both cell types. Interestingly, treatment with all perilla seed extracts and DF significantly reduced nitrite amounts in comparison to the LPS-treated group (*p* < 0.05). Additionally, SFE-PS demonstrated the strongest inhibitory effect on NO production, significantly outperforming MAC-PS and SC-PS. Notably, the NO suppression by SFE-PS was comparable to that of DF in both macrophages and HFDPCs. These results suggested that SFE-PS exhibited anti-inflammatory efficacy similar to that of DF.

Previous research has indicated that NO concentrations in hair follicles actively contribute to the regulation of hair regeneration throughout the anagen phase and may contribute to hair loss during the telogen phase [45]. Among the extracts tested, SFE-PS exhibited the strongest NO inhibitory activity, followed by MAC-PS and SC-PS. Although SFE-PS and SC-PS demonstrated a similar overall fatty acid composition, including high levels of linoleic acid, α-linolenic acid, and oleic acid—bioactive compounds known for their antioxidative and anti-inflammatory effects—only SFE-PS contained a significant amount of caffeic acid (12.80 ± 0.00 μg/g extract), which was not detected in SC-PS [37]. The combination of fatty acids and phenolic compounds, particularly caffeic acid, may have contributed to the superior NO inhibition observed in SFE-PS [37].

In contrast, MAC-PS showed no detectable levels of fatty acids but exhibited a moderate inhibitory effect on NO production, which was likely attributable to its rich phenolic profile. Notably, MAC-PS was the only extract in which polyphenols such as epicatechin, caffeic acid, and gallic acid were identified; these compounds were absent in both SFE-PS and SC-PS. These polyphenols have been reported to suppress inflammatory mediators, including NO, by downregulating iNOS expression and reducing oxidative stress. Therefore, the NO-inhibitory effects of MAC-PS were presumably mediated by its phenolic constituents rather than by lipid-based bioactivities [46].

As described, SFE-PS exhibited the strongest NO inhibition, followed by MAC-PS and SC-PS. SFE-PS uniquely contained caffeic acid, a phenolic compound with a catechol moiety (ortho-dihydroxybenzene) that facilitates radical scavenging and iNOS suppression through hydrogen donation and resonance stabilization [47]. Although MAC-PS also contained polyphenols such as epicatechin and gallic acid, which possess anti-inflammatory potential, these compounds may have limited cellular uptake due to their larger molecular size and lower lipophilicity [48,49]. Importantly, both SFE-PS and SC-PS presented a full profile of bioactive lipophilic compounds, including *α*-, *β*-, *γ*-, and *δ*-tocopherols and various unsaturated fatty acids such as linoleic, α-linolenic, and oleic acids—all of which were not detected in MAC-PS. These lipophilic antioxidants are known to enhance membrane protection and modulate inflammatory signaling pathways [50]. Therefore, the superior anti-inflammatory activity of SFE-PS may result not only from its phenolic composition but also from the combined presence and possible synergistic effects of tocopherols and unsaturated fatty acids. In contrast, the absence of these lipophilic compounds in MAC-PS likely limited its overall anti-inflammatory potential despite its diverse polyphenolic profile.

These findings revealed that fatty acids predominantly contributed to NO suppression in SFE-PS and SC-PS, whereas the effect exhibited by MAC-PS was likely due to its polyphenolic content. This finding highlighted the importance of both fatty acids and polyphenols in modulating inflammatory responses relevant to hair follicle maintenance and regeneration [46,51]. Nevertheless, it should be noted that these anti-inflammatory observations were based on a single fixed concentration (0.125 mg/mL), and further dose–response analysis is required to determine and quantify the effectiveness of each extract.

### 3.4. Effect of Perilla Seed Extracts on Antioxidant Activities

Oxidative stress can induce lipid peroxidation, a process in which ROS react with polyunsaturated fatty acids in cellular membranes. This oxidative damage disrupts membrane structure and function, leading to cellular injury. MDA, a reliable biomarker of lipid peroxidation and oxidative stress, is commonly used to evaluate the lipid peroxidation [52].

**Figure 3:**
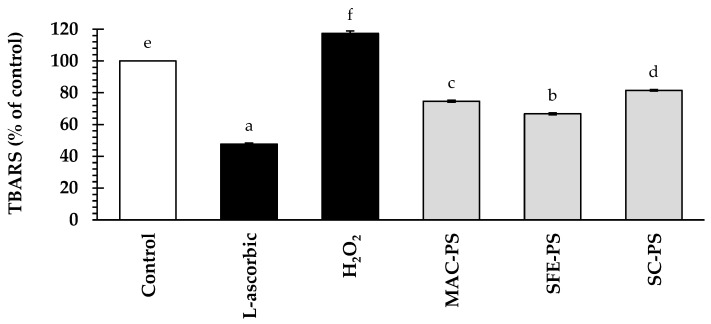
The effect of perilla seed extracts on TBARS levels in hydrogen peroxide (H_2_O_2_)-induced HFDPCs evaluated in comparison with the negative control (H_2_O_2_-induced without pre-treatment), standard treatment (L-ascorbic acid), and untreated control, at 0.125 mg/mL. Data are reported as mean ± SD for each sample. Statistical comparisons are applied through a one-way ANOVA followed by Tukey’s post hoc test. Distinct lowercase letters (a–f) represent statistically significant differences among groups in each assay (*p* < 0.05). Abbreviations: MAC-PS, macerated perilla seed extract; SFE-PS, supercritical fluid-extracted perilla seed extract; SC-PS, screw-pressed perilla seed extract.

To investigate the antioxidant potential of perilla seed extracts, HFDPCs were pre-treated with perilla seed extracts or L-ascorbic acid (a positive control) at a concentration of 0.125 mg/mL prior to H_2_O_2_ exposure (Figure 3). The results showed that H_2_O_2_ treatment notably increased TBARS amounts to 117.45 ± 0.50% of the control (*p* < 0.05). Interestingly, all perilla seed extracts significantly reduced TBARS levels, ranging from 67.75 ± 0.61% to 81.12 ± 0.52% of the control, indicating a decrease in oxidative stress. Although L-ascorbic acid exhibited the strongest suppression (47.74 ± 0.59% of the control), perilla seed extracts still demonstrated notable antioxidant activity as a potential natural alternative.

Among the tested extracts, SFE-PS exhibited the greatest suppression of TBARS levels, followed by MAC-PS and SC-PS, respectively. This superior antioxidant property may be attributed to the synergistic effect of its abundant fatty acids and the presence of caffeic acid in the SFE-PS. Although SC-PS was rich in fatty acids, it lacked caffeic acid. On the other hand, MAC-PS contained various polyphenols but no detectable fatty acids [53]. The variation in antioxidant capacity among the extracts can be described by the specific bioactive compounds found in each extract. Polyunsaturated fatty acids (such as linoleic acid and *α*-linolenic acid), and tocopherols have been reported to reduce oxidative stress by preserving cell membrane integrity. Also, polyphenols such as caffeic acid have been reported to reduce oxidative damage via neutralizing free radicals and activating cellular mechanisms [53,54].

### 3.5. Effect of Perilla Seed Extracts on Genes Associated with Hair Loss and Hair Growth

Hair follicle development relies on coordinated interactions among multiple cell types, such as HFDPCs, fibroblasts, and epithelial cells. Among these, HFDPCs are recognized as key regulators that govern follicle morphogenesis and promote the initiation and maintenance of the hair growth cycle. This cycle comprises four distinct phases—the anagen marked by active growth, catagen as a transitional phase, telogen as the resting stage, and exogen where hair is naturally shed—and each stage is modulated through complex cellular signaling networks [55].

A major factor contributing to AGA is the dysregulation of androgen and TGF-β signaling pathways. In particular, DHT—a potent androgen derived from testosterone via the action of SRD5A enzymes—has been shown to accelerate the transition from anagen to catagen, leading to hair follicle miniaturization and eventual hair loss [1]. Three *SRD5A* isoforms—*SRD5A1*, *SRD5A2*, and *SRD5A3*—are expressed in hair follicles and contribute to increased DHT levels in androgen-sensitive regions of the scalp [56]. Moreover, DHT induces *TGF-β* expression in HFDPCs, which subsequently triggers apoptotic pathways in follicular epithelial cells. TGF-β is particularly known for accelerating the transition from the anagen to the catagen phase, thereby contributing to hair loss [57]. This study examined the inhibitory effects of perilla seed extracts and standard treatments (dutasteride, finasteride, and minoxidil at 0.125 mg/mL) on *SRD5A1–3* expression in human prostate cancer cells and HFDPCs, alongside their effects on the downregulation of *TGF-β* expression in HFDPCs.

**Figure 4:**
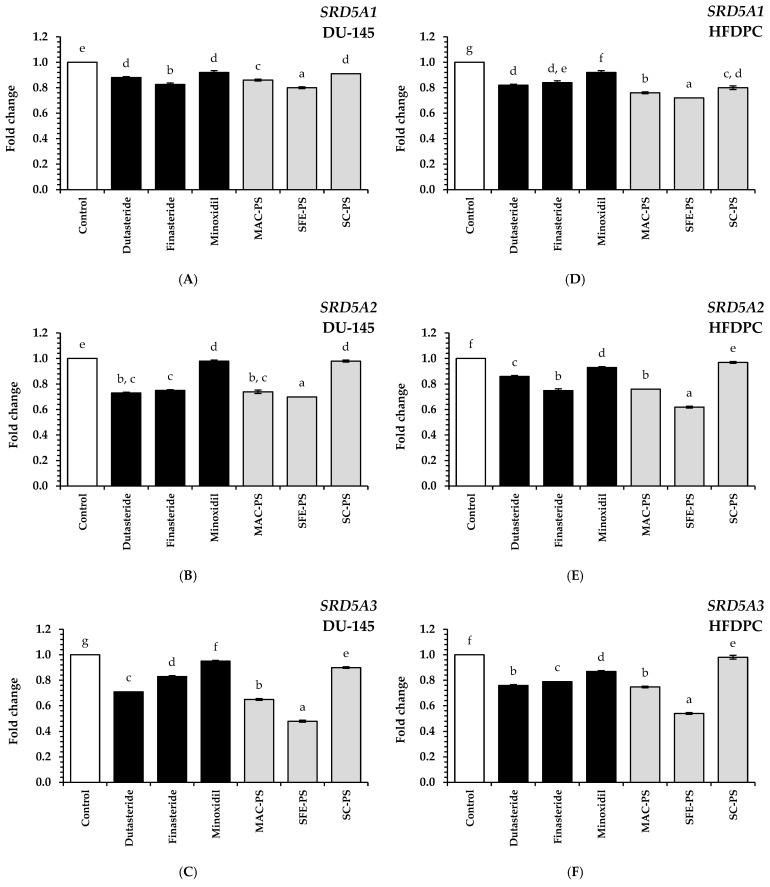
Gene expression related to the androgen pathway is analyzed following treatment with perilla seed extracts: (**A**) *SRD5A1*, (**B**) *SRD5A2*, and (**C**) *SRD5A3* in DU-145 cells, and (**D**) *SRD5A1*, (**E**) *SRD5A2*, and (**F**) *SRD5A3* in HFDPCs. These effects are compared to those of conventional therapeutic treatments (dutasteride, finasteride, and minoxidil) at 0.125 mg/mL. Data are reported as mean ± SD for each sample. Statistical comparisons are applied through a one-way ANOVA followed by Tukey’s post hoc test. Distinct lowercase letters (a–g) represent statistically significant differences among groups in each assay (*p* < 0.05). Abbreviations: MAC-PS, macerated perilla seed extract; SFE-PS, supercritical fluid-extracted perilla seed extract; SC-PS, screw-pressed perilla seed extract.

As illustrated in Figure 4, treatment with perilla seed extracts markedly downregulated the mRNA levels of *SRD5A1–3* in both DU-145 and dermal papilla cell lines when compared to the untreated control group (*p* < 0.05). Moreover, these extracts demonstrated significantly greater suppression of these genes than the standard treatments, including dutasteride, finasteride, and minoxidil (*p* < 0.05). Among the tested extracts, SFE-PS exhibited the most potent inhibitory effect on *SRD5A1–3* gene expression. In DU-145 cells treated with SFE-PS, the fold changes for *SRD5A1*, *SRD5A2*, and *SRD5A3* were 0.80 ± 0.01, 0.70 ± 0.01, and 0.48 ± 0.01, respectively. Similarly, in HFDPCs treated with SFE-PS, the fold changes were 0.72 ± 0.01, 0.62 ± 0.01, and 0.54 ± 0.01, respectively.

Notably, the gene suppression activity of SFE-PS was 1.10, 1.25, and 1.50 times greater than that of dutasteride, finasteride, and minoxidil, respectively, based on the average expression levels of *SRD5A1–3* across both cell types (*p* < 0.05). This superior activity may be attributed to the high content of bioactive compounds in the SFE-PS extract, particularly tocopherols, polyphenols (especially caffeic acid), and fatty acids such as *α*-linolenic acid, linoleic acid, oleic acid, and palmitic acid. Previous studies have reported that these compounds can inhibit SRD5A enzyme activity, supporting the current findings [37].

When comparing extraction methods, SFE-PS showed significantly greater suppression of *SRD5A1–3* gene and exhibited enhanced biological responses than MAC-PS and SC-PS (*p* < 0.05). These results were consistent with previous antioxidant and anti-inflammatory assays, in which SFE-PS consistently demonstrated higher levels of key bioactive compounds. Notably, its rich content of unsaturated fatty acids—particularly *α*-linolenic acid, linoleic acid, and oleic acid—combined with the presence of caffeic acid and tocopherols may synergistically contribute to the considerable repression of *SRD5A1–3* gene expression observed in this study [24,37].

TGF-β participates in modulating the catagen phase during hair follicle cycling, particularly in terms of androgen-related hair loss. Previous studies have demonstrated that DHT stimulates the production of TGF-β in HFDPCs, which subsequently suppresses epithelial cell proliferation and induces apoptosis [3]. To further explore the role of TGF-β in androgen-induced hair follicle regression, this study evaluated the gene expression levels of *TGF-β1* in HFDPCs following treatment with perilla seed extracts and standard treatments.

**Figure 5:**
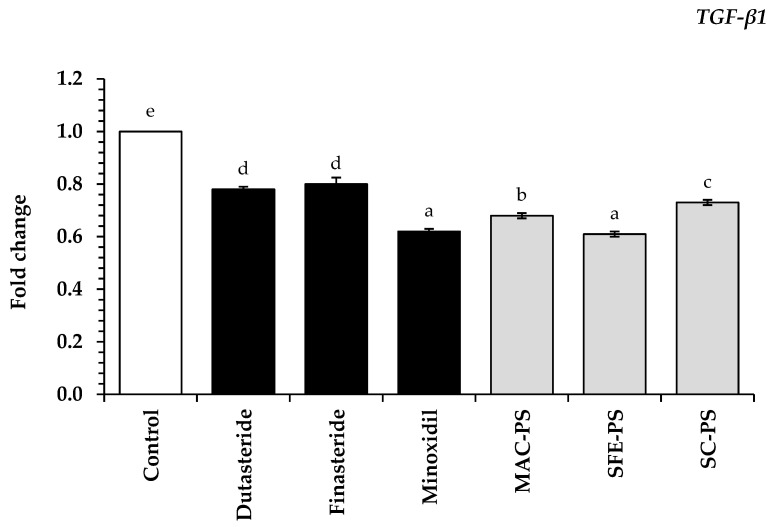
The expression of transforming growth factor-beta 1 (*TGF-β1*) gene is analyzed following treatment with perilla seed extracts in HFDPCs. These effects are compared with those of conventional therapeutic treatments (dutasteride, finasteride, and minoxidil) at a concentration of 0.125 mg/mL. Data are reported as mean ± SD for each sample. Statistical comparisons are applied through a one-way ANOVA followed by Tukey’s post hoc test. Distinct lowercase letters (a–e) represent statistically significant differences among groups in each assay (*p* < 0.05). Abbreviations: MAC-PS, macerated perilla seed extract; SFE-PS, supercritical fluid-extracted perilla seed extract; SC-PS, screw-pressed perilla seed extract.

As illustrated in Figure 5, all perilla seed extracts significantly downregulated *TGF-β1* gene expression in HFDPCs compared to the untreated control group (*p* < 0.05). Notably, the SFE-PS extract demonstrated no significant difference in *TGF-β1* suppression compared to minoxidil, a standard hair loss treatment (*p* > 0.05). Interestingly, all perilla seed extracts significantly reduced *TGF-β1* expression relative to dutasteride and finasteride (*p* < 0.05). Among the tested extracts, SFE-PS demonstrated the most marked inhibitory effect (0.61 ± 0.01), followed by MAC-PS (0.68 ± 0.01) and SC-PS (0.73 ± 0.01). These findings were in accordance with the proposed anti-androgenic effects of perilla seed extracts, which have been linked to *SRD5A* inhibition and the modulation of *TGF-β1* expression [58,59].

The suppression of *TGF-β1* may be linked to the downregulation of *SRD5A1–3* genes as these enzymes catalyze the conversion of testosterone to DHT—a known inducer of TGF-β signaling in HFDPCs. The perilla seed extracts evaluated in this study may have indirectly attenuated DHT-mediated *TGF-β1* induction, leading to disruption of the pro-apoptotic signaling cascade responsible for hair follicle regression. This inhibition of both upstream (*SRD5A*) and downstream (*TGF-β1*) targets within the signaling pathway provides preliminary evidence of the comprehensive anti-androgenic activity of perilla seed extracts and suggests their potential role in preventing hair loss.

Based on these findings, clinical trials involving formulations containing perilla seed extracts are planned for future investigation to further validate its efficacy in the treatment of androgenetic alopecia.

## 4. Conclusions

This study demonstrated the critical role of the extraction method in influencing both the chemical composition and the in vitro biological activity of perilla seed extracts. Among the techniques examined, SFE yielded extracts enriched with unsaturated fatty acids and tocopherols, while MAC uniquely retained a broader spectrum of polyphenols. Standardized analytical techniques, including LC-MS for polyphenol profiling, GC-FID for fatty acid analysis, and HPLC for tocopherol quantification, were employed to explore the relationship between specific bioactive constituents and their in vitro biological effects related to hair health. The SFE extract showed consistent in vitro responses, including promotion of dermal papilla cell proliferation, reduction in oxidative stress and inflammation, and suppression of key AGA-related genes (*SRD5A* and *TGF-β1*). These findings emphasize the capability of SFE as an efficient technique for obtaining bioactive-rich extracts. Whilst promising, these results represent early-stage evidence and warrant further in vivo studies to confirm their relevance in hair loss prevention or scalp care applications. Although the in vitro findings are promising, further in vivo investigations are warranted to validate their potential application in hair loss prevention and scalp health care.

## Figures and Tables

**Table 1:** Forward and reverse primer sequences applied for gene expression analysis by RT-PCR.

Target Pathway	Primers	Gene Bank No.	Sequences	Primer Sequences (5’ to 3’)	Annealing Temperature (°C)
Internal control	*GAPDH*	NM_001289745.3	Sense Antisense	GGAAGGTGAAGGTCGGAGTC CTCAGCCTTGACGGTGCCATG	55
5*α*-reductase	*SRD5A1*	NM_001047.4	Sense Antisense	AGCCATTGTGCAGTGTATGC AGCCTCCCCTTGGTATTTTG	52
	*SRD5A2*	NM_000348.4	Sense Antisense	TGAATACCCTGATGGGTGG CAAGCCACCTTGTGGAATC	52
	*SRD5A3*	NM_024592.5	Sense Antisense	TCCTTCTTTGCCCAAACATC TCCTTCTTTGCCCAAACATC	50
Transforming growth factor beta 1	*TGF-β1*	NM_00600.7	Sense Antisense	AACCCACAACGAAATCTATG CTTTTAACTTGAGCCTCAGC	58

**Table 2:** Extraction yield, phytochemical compositions, and antioxidant activities of perilla seed extracts.

Results		Extracts		
		MAC-PS	SFE-PS	SC-PS
Extraction yield (%)		42.34 ± 0.11	33.93 ± 0.28	22.46 ± 0.20
Total phenolic content (mg GAE/g extract)		23.07 ± 0.32	8.22 ± 0.73	1.17 ± 0.39
Polyphenols (μg/g extract)	Gallic acid	8.27 ± 0.01	ND	ND
	Epicatehchin	29.14 ± 4.36	ND	ND
	Caffeic acid	17.54 ± 0.28 ^b^	12.80 ± 0.00 ^a^	ND
	Epicathechin gallate	8.69 ± 0.78	ND	ND
	Naringin	7.32 ± 0.11	ND	ND
	Rosmarinic acid	16.35 ± 0.05	ND	ND
	*o*-Coumaric acid	6.24 ± 0.33	ND	ND
	*p*-Coumaric acid	13.23 ± 0.01	ND	ND
	Quercetin	7.58 ± 0.12	ND	ND
Tocopherol (μg/g extract)	*α*-Tocopherol	ND	20.58 ± 1.87 ^a^	25.68 ± 0.36 ^b^
	*β*- and *γ*-Tocopherol	ND	485.84 ± 0.84 ^a^	513.16 ± 3.77 ^b^
	*δ*-Tocopherol	ND	13.40 ± 0.50 ^a^	14.08 ± 0.02 ^b^
Antioxidant activity	ABTS radical scavenging (%)	85.72 ± 0.96 ^c^	38.30 ± 0.57 ^b^	28.89 ± 1.82 ^a^
	DPPH radical scavenging (%)	75.70 ± 2.71 ^c^	45.29 ± 0.72 ^b^	32.19 ± 0.90 ^a^
	FRAP (mM Fe^2+^/g extract)	68.32 ± 2.11 ^a^	121.21 ± 2.72 ^c^	92.38 ± 1.14 ^b^
	FTC (mg TEAC/g extract)	9.85 ± 1.36 ^a^	17.38 ± 1.02 ^b^	19.53 ± 1.04 ^c^

Note: Results are reported as mean ± standard deviation (SD) for each extract. Statistical tests were applied through one-way ANOVA followed by Tukey’s post hoc test. Distinct lowercase letters (a–c) represent statistically significant differences among groups in each assay (*p* < 0.05). Abbreviations: mg GAE/g extract, milligrams of gallic acid equivalent per gram of extract; μg/g extract, micrograms per gram of extract; mM Fe^2+^/g extract, millimoles of ferrous ion equivalents per gram of extract; mg TEAC/g extract, milligrams of Trolox equivalent antioxidant capacity per gram of extract; MAC-PS, macerated perilla seed extract; SFE-PS, supercritical fluid-extracted perilla seed extract; SC-PS, screw-pressed perilla seed extract; ND, not detected.

**Table 3:** Fatty acid composition (%) of perilla seed extracts obtained using maceration (MAC-PS), supercritical fluid extraction (SFE-PS), and screw extraction (SC).

Classification	Name	MAC-PS	SFE-PS	SC-PS
Saturated Fatty Acids	Pentadecanoic acid	ND	0.01 ^a^	0.01 ^a^
	Palmitic acid	ND	6.83 ^a^	6.88 ^b^
	Heptadecanoic acid	ND	0.14 ^a^	0.14 ^a^
	Stearic acid	ND	2.88 ^a^	2.90 ^b^
	Arachidic acid	ND	0.18 ^a^	0.18 ^a^
	Behenic acid	ND	0.02 ^a^	0.03 ^b^
	Heneicosanoic acid	ND	0.05 ^a^	0.05 ^a^
	Butyric acid	ND	0.76 ^a^	0.86 ^b^
	Myristic acid	ND	0.02 ^a^	0.02 ^a^
Monounsaturated Fatty Acids	Palmitoleic acid	ND	0.01 ^a^	0.01 ^a^
	Heptadecenoic acid	ND	0.01 ^a^	0.01 ^a^
	Oleic acid	ND	11.75 ^a^	11.74 ^a^
	Gondoic acid	ND	0.04 ^a^	0.07 ^b^
	Erucic acid	ND	0.03 ^b^	0.01 ^a^
	Nervonic acid	ND	0.01 ^a^	0.01 ^a^
Polyunsaturated Fatty Acids	Trans-Linoleic acid	ND	0.07 ^b^	0.06 ^a^
	Linoleic acid	ND	17.45 ^b^	17.36 ^a^
	*γ*-Linolenic acid	ND	0.21 ^a^	0.21 ^a^
	*α*-Linolenic acid	ND	59.43 ^b^	59.23 ^a^
	Eicosadienoic acid	ND	0.01 ^a^	0.02 ^b^
	Dihomo-*γ*-linolenic acid	ND	0.02 ^a^	0.02 ^a^
	Eicosatrienoic acid	ND	0.01 ^a^	0.01 ^a^
	Eicosapentaenoic acid	ND	0.02 ^a^	0.02 ^a^
	Docosahexaenoic acid	ND	0.01 ^a^	0.01 ^a^

Note. Results are reported as percentages (%) of the total fatty acid content for each extraction method. Statistical comparisons were applied through a one-way ANOVA followed by Tukey’s post hoc test. Distinct lowercase letters (a–b) indicate statistically significant differences between groups (*p* < 0.05). The MAC-PS group was excluded from statistical analysis due to all values being not detected (ND). Abbreviations: MAC-PS, macerated perilla seed extract; SFE-PS, supercritical fluid-extracted perilla seed extract; SC-PS, screw-pressed perilla seed extract; ND, not detected.

## Data Availability

The original contributions presented in this study are included in the article/Supplementary Materials. Further inquiries can be directed to the corresponding author.

## Supplementary Materials

The following supporting information can be downloaded at: https://www.mdpi.com/article/10.3390/foods14152583/s1, Figure S1: HPLC chromatograms of (a) polyphenol standards and polyphenol profiles of (b) MAC-PS, (c) SFE-PS, and (d) SC-PS extracts; Figure S2: HPLC chromatograms of (a) tocopherol standards and tocopherol composition in (b) MAC-PS, (c) SFE-PS, and (d) SC-PS extracts; Figure S3: GC-FID chromatograms of (a) fatty acid standards and fatty acid profiles in (b) MAC-PS, (c) SFE-PS, and (d) SC-PS extracts; Table S1: Cell viability responses of RAW 264.7, hTERT fibroblast, HaCaT keratinocyte, DU-145 cells, and HFDPCs to perilla seed extracts at all tested concentrations.
