# Supplementary material for: Anti-Hair Loss Potential of Perilla Seed Extracts: In Vitro Molecular Insights from Supercritical Fluid Extraction
Source: Foods. 2025 Jul 23;14(15):2583. doi: 10.3390/foods14152583 (PMC12346375; doi:10.3390/foods14152583)
Supplement: Supplementary file 1 [file foods-14-02583-s001.zip › foods-3698237-supplementary.pdf]

Article

Anti-Hair Loss Potential of Perilla Seed Extracts: *In Vitro* Molecular Insights from Supercritical Fluid Extraction

Supplementary Information

<Sample Information>

Sample Name	: 1		
Sample ID	: 1		
Data Filename	: 1.lcd		
Method Filename	: polyphenol_method.lcm		
Batch Filename	: 3.lcb		
Vial #	: 1-97	Sample Type	: Unknown
Injection Volume	: 10 uL		
Date Acquired	: 27/2/2568 13:17:25	Acquired by	: System Administrator
Date Processed	: 27/2/2568 16:03:32	Processed by	: System Administrator

<Chromatogram>

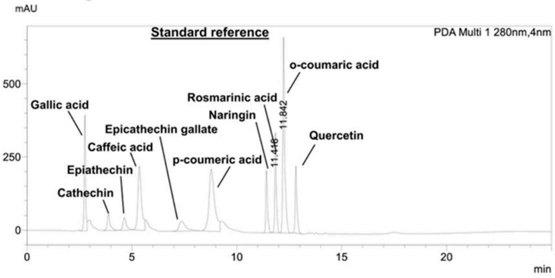

<Peak Table>

PDA Ch1 280nm

Peak#	Ret. Time	Area	Height	Conc.	Unit	Mark	Name
1	3.096	17878	2742	0.000		M	
2	4.014	26629	4239	0.000	mg/L	M	Epicatehchin
3	5.188	39546	5933	0.000		M	
4	6.890	18704	1649	0.000	mg/L	M	Epicathechin gallate
5	8.109	32153	2346	0.000	mg/L	M	P-coumeric
6	9.651	8831	654	0.000		M	
7	10.504	5935	423	0.000		M	
8	12.841	5134	306	0.000	mg/L	M	Quercetin
9	13.599	9839	555	0.000		M	
10	17.984	2890	120	0.000		M	
11	21.950	22495	570	0.000		M	
Total		190035	19536				

(**a**)

<Sample Information>

Sample Name	: mix A-1		
Sample ID	: mix A-1		
Data Filename	: mix A-1.lcd		
Method Filename	: polyphenol_method.lcm		
Batch Filename	: 1.lcb		
Vial #	: 1-61	Sample Type	: Unknown
Injection Volume	: 10 uL		
Date Acquired	: 19/2/2568 18:05:50	Acquired by	: System Administrator
Date Processed	: 28/2/2568 16:33:42	Processed by	: System Administrator

<Chromatogram>

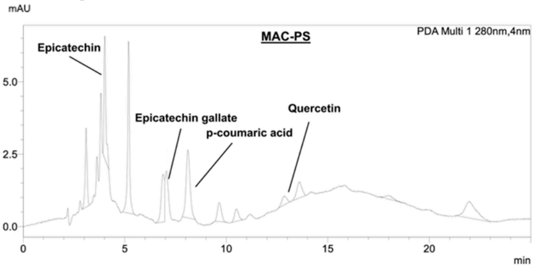

<Peak Table>

PDA Ch1 280nm

Peak#	Ret. Time	Area	Height	Conc.	Unit	Mark	Name
1	2.758	1780122	396200	0.000	mg/L	M	Gallic acid
2	3.883	534805	58357	0.000	mg/L	M	Cathechin
3	4.641	510812	43165	0.000	mg/L	M	Epicatehchin
4	5.353	2624547	219048	0.000	mg/L	M	Caffeic acid
5	7.364	769826	34478	0.000	mg/L	M	Epicathechin gallate
6	8.785	3985499	212100	0.000	mg/L	M	P-coumeric
7	11.418	1171740	212786	0.000	mg/L	M	Naringin
8	11.842	1725123	342440	0.000	mg/L	M	Rosmarinic acid
9	12.230	4133613	669623	0.000	mg/L	V M	O-coumeric
10	12.819	1312672	230009	0.000	mg/L	M	Quercetin
Total		18548761	2418207				

(**b**)

<Sample Information>

Sample Name	: 9		
Sample ID	: 9		
Data Filename	: 9.lcd		
Method Filename	: polyphenol_method.lcm		
Batch Filename	: 3.lcb		
Vial #	: 1-105	Sample Type	: Unknown
Injection Volume	: 10 uL		
Date Acquired	: 28/2/2568 15:35:42	Acquired by	: System Administrator
Date Processed	: 28/2/2568 16:00:44	Processed by	: System Administrator

<Chromatogram>

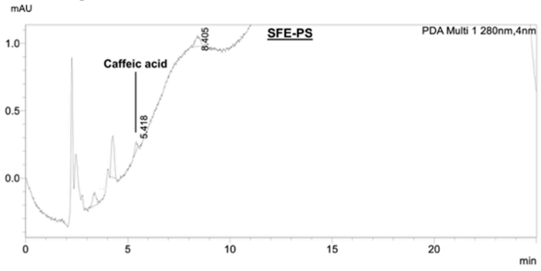

<Peak Table>

PDA Ch1 280nm

Peak#	Ret. Time	Area	Height	Conc.	Unit	Mark	Name
1	3.382	1158	101	0.000		M	
2	4.267	3001	307	0.000		M	
3	5.418	522	70	0.000	mg/L	M	Caffeic acid
4	8.405	1286	82	0.000		M	
Total		5967	560				

(**c**)

<Sample Information>

Sample Name	: 6-1		
Sample ID	: 6-1		
Data Filename	: 6-1.lcd		
Method Filename	: polyphenol_method.lcm		
Batch Filename	: 3.lcb		
Vial #	: 1-102	Sample Type	: Unknown
Injection Volume	: 10 uL		
Date Acquired	: 28/2/2568 12:11:10	Acquired by	: System Administrator
Date Processed	: 28/2/2568 12:36:13	Processed by	: System Administrator

<Chromatogram>

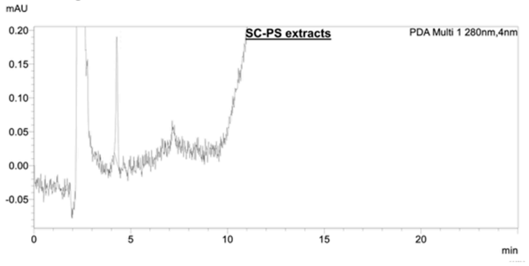

<Peak Table>

PDA Ch1 280nm

Peak#	Ret. Time	Area	Height	Conc.	Unit	Mark	Name
1	4.280	1099	185	0.000			
Total		1099	185				

(**d**)

Figure S1. HPLC chromatograms of (**a**) polyphenol standards and polyphenol profiles of (**b**) MAC-PS, (**c**) SFE-PS, and (**d**) SC-PS extracts.

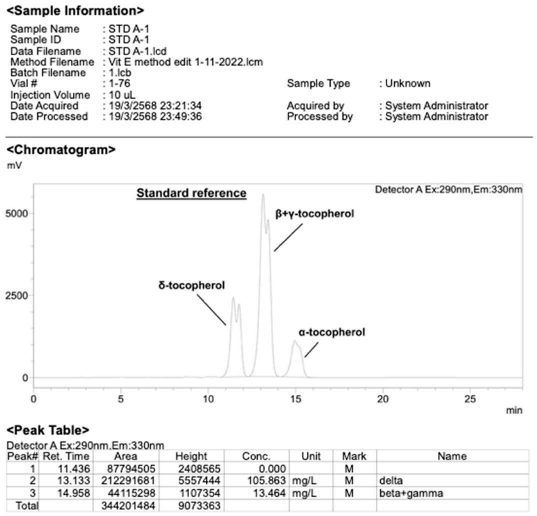

Peak#	Ret. Time	Area	Height	Conc.	Unit	Mark	Name
1	11.436	87794505	2408565	0.000		M	
2	13.133	212291681	5557444	105.863	mg/L	M	delta
3	14.958	44115298	1107354	13.464	mg/L	M	beta+gamma
Total		344201484	9073363				

(**a**)

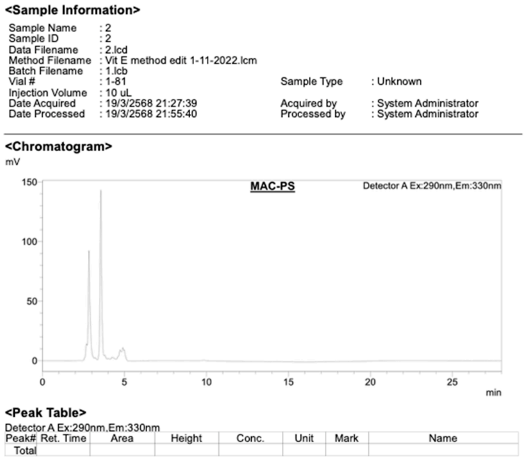

Peak#	Ret. Time	Area	Height	Conc.	Unit	Mark	Name
Total							

(**b**)

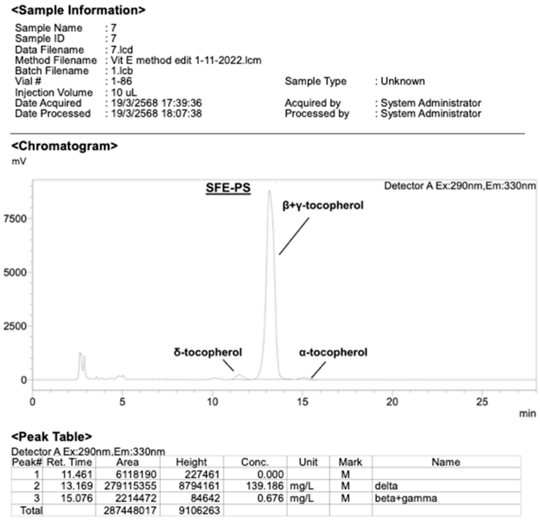

Peak#	Ret. Time	Area	Height	Conc.	Unit	Mark	Name
1	11.461	6118190	227461	0.000		M	
2	13.169	279115355	8794161	139.186	mg/L	M	delta
3	15.076	2214472	84642	0.676	mg/L	M	beta+gamma
Total		287448017	9106263				

(**c**)

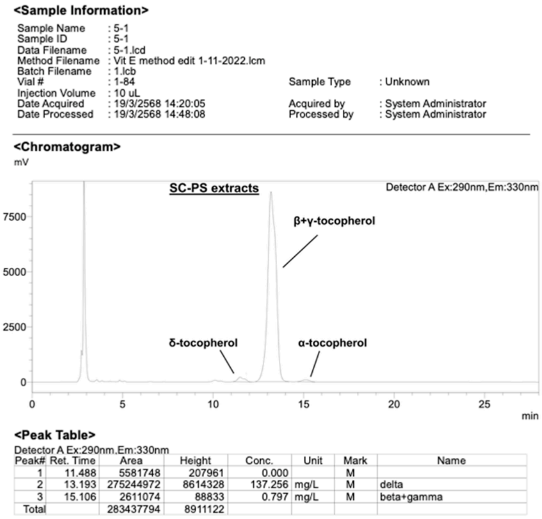

Peak#	Ret. Time	Area	Height	Conc.	Unit	Mark	Name
1	11.488	5581748	207961	0.000		M	
2	13.193	275244972	8614328	137.256	mg/L	M	delta
3	15.106	2611074	88833	0.797	mg/L	M	beta+gamma
Total		283437794	8911122				

(**d**)

Figure S2. HPLC chromatograms of (**a**) tocopherol standards and tocopherol composition in (**b**) MAC-PS, (**c**) SFE-PS, and (**d**) SC-PS extracts.

<Sample Information>

Sample Name	: STD 1
Sample ID	: STD 1
Data Filename	: STD 1.gcd
Method Filename	: FFA_method 10-9-2566 rate1.5 inj2.gcm
Batch Filename	: 1.gcb
Vial #	: 26
Injection Volume	: 2 uL
Date Acquired	: 11/11/2567 14:37:48
Date Processed	: 12/11/2567 13:14:32
Sample Type	: Unknown
Acquired by	: System Administrator
Processed by	: System Administrator

<Chromatogram>

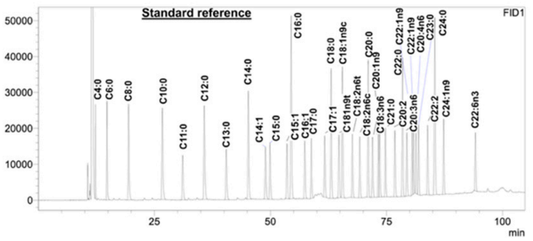

<Peak Table>

FID1

Peak#	Ret. Time	Area	Height	Conc.	Unit	Mark	Name
1	12.297	157362	26382	0.000	ppm		C4:0
2	14.775	259672	27928	0.000	ppm		C6:0
3	19.442	348980	26738	0.000	ppm		C8:0
4	26.715	387530	25469	0.000	ppm		C10:0
5	31.087	194970	12449	0.000	ppm		C11:0
6	35.743	416515	26696	0.000	ppm	M	C12:0
7	40.488	214035	14044	0.000	ppm		C13:0
8	45.246	441948	30111	0.000	ppm		C14:0
9	48.947	218302	14538	0.000	ppm		C14:1
10	49.896	223101	15760	0.000	ppm		C15:0
11	53.546	220883	15331	0.000	ppm	M	C15:1
12	54.466	695562	50958	0.000	ppm		C16:0
13	57.391	226112	16041	0.000	ppm		C16:1
14	58.816	227207	17138	0.000	ppm		C17:0
15	61.680	234513	17111	0.000	ppm		C17:1
16	63.081	471645	36196	0.000	ppm		C18:0
17	64.765	236202	17520	0.000	ppm	M	C18:1n9t
18	65.492	483635	36416	0.000	ppm	M	C18:1n9c
19	67.646	233671	17702	0.000	ppm		C18:2n6t
20	69.238	238261	17673	0.000	ppm		C18:2n6c
21	71.065	472456	38290	0.000	ppm		C20:0
22	71.988	228072	16807	0.000	ppm		C18:3n6

(**a**)

<Sample Information>

Sample Name	: 2-1
Sample ID	: 2-1
Data Filename	: 2-1.gcd
Method Filename	: FFA_method 10-9-2566 rate1.5 inj4.gcm
Batch Filename	: 1.gcb
Vial #	: 142
Injection Volume	: 4 uL
Date Acquired	: 27/2/2568 22:31:04
Date Processed	: 3/3/2568 10:59:17
Sample Type	: Unknown
Acquired by	: System Administrator
Processed by	: System Administrator

<Chromatogram>

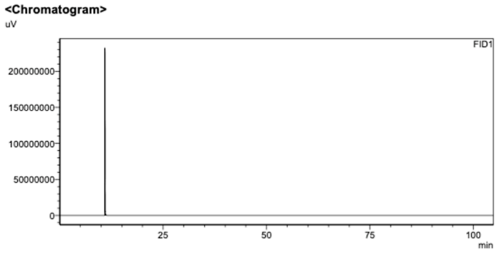

<Peak Table>

FID1

Peak#	Ret. Time	Area	Height	Conc.	Unit	Mark	Name
Total							

(**b**)

<Sample Information>

Sample Name	: 7
Sample ID	: 7
Data Filename	: 7.gcd
Method Filename	: FFA_method 10-9-2566 rate1.5 inj4.gcm
Batch Filename	: 1.gcb
Vial #	: 147
Injection Volume	: 4 uL
Date Acquired	: 28/2/2568 15:00:02
Date Processed	: 28/2/2568 16:44:54
Sample Type	: Unknown
Acquired by	: System Administrator
Processed by	: System Administrator

<Chromatogram>

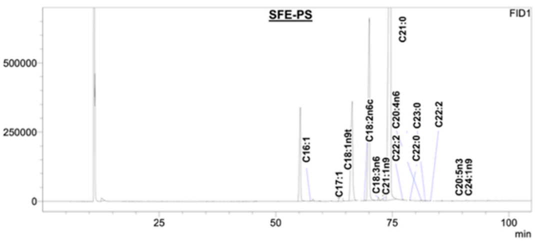

<Peak Table>

FID1

Peak#	Ret. Time	Area	Height	Conc.	Unit	Mark	Name
1	12.650	295497	10950	0.000		M	
2	45.796	14081	1037	0.000		M	
3	50.428	4437	395	0.000		M	
4	55.239	5847643	340147	0.000		M	
5	57.432	12040	1009	0.000	ppm	M	C16:1
6	57.975	119231	6442	0.000		M	
7	59.372	30118	1697	0.000	ppm	M	C17:0
8	61.946	9400	797	0.000	ppm	M	C17:1
9	64.190	2487827	106476	0.000	ppm	M	C18:1n9t
10	66.421	10579908	358576	0.000		M	
11	67.992	2623	277	0.000	ppm	M	C18:2n6t
12	69.026	56410	3415	0.000	ppm	M	C18:2n6c
13	70.082	14458812	660057	0.000		M	
14	72.004	149598	11404	0.000	ppm	M	C18:3n6
15	72.897	164529	6133	0.000	ppm	M	C20:1n9
16	74.653	49263140	1691890	0.000	ppm	M	C21:0
17	77.181	34670	2648	0.000	ppm	M	C20:2
18	78.309	9486	898	0.000	ppm	M	C22:0
19	78.774	17118	1709	0.000	ppm	M	C22:0
20	81.206	42753	2570	0.000	ppm	M	C20:4n6
21	81.693	1481	165	0.000	ppm	M	C23:0
22	82.029	19661	1288	0.000	ppm	M	C23:0

(**c**)

<Sample Information>

Sample Name	: 6-1
Sample ID	: 6-1
Data Filename	: 6-1.gcd
Method Filename	: FFA_method 10-9-2566 rate1.5 inj4.gcm
Batch Filename	: 1.gcb
Vial #	: 146
Injection Volume	: 4 uL
Date Acquired	: 28/2/2568 13:10:10
Date Processed	: 3/3/2568 14:06:46
Sample Type	: Unknown
Acquired by	: System Administrator
Processed by	: System Administrator

<Chromatogram>

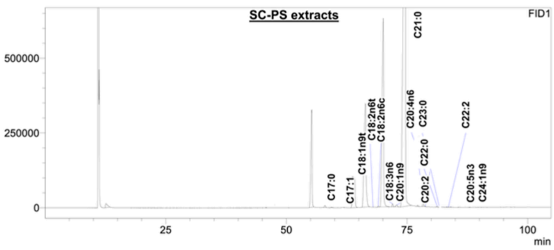

<Peak Table>

FID1

Peak#	Ret. Time	Area	Height	Conc.	Unit	Mark	Name
1	12.599	413921	13351	0.000		M	
2	45.778	11245	886	0.000		M	
3	50.413	4211	380	0.000		M	
4	55.229	5421706	325979	0.000		M	
5	57.980	118246	6725	0.000		M	
6	59.360	26726	1639	0.000	ppm	M	C17:0
7	61.908	7154	607	0.000	ppm	M	C17:1
8	64.165	2271252	100229	0.000	ppm	M	C18:1n9t
9	66.388	9910007	346113	0.000		M	
10	67.957	1438	173	0.000	ppm	M	C18:2n6t
11	69.020	53070	3326	0.000	ppm	M	C18:2n6c
12	70.068	13534713	630717	0.000		M	
13	71.987	135766	10299	0.000	ppm	M	C18:3n6
14	72.888	166392	6320	0.000	ppm	M	C20:1n9
15	74.644	47289321	1655123	0.000	ppm	M	C21:0
16	77.201	46308	3321	0.000	ppm	M	C20:2
17	78.350	218813	17034	0.000	ppm	M	C22:0
18	78.783	9368	1174	0.000	ppm	M	C22:0
19	81.245	43727	2518	0.000	ppm	M	C20:4n6
20	81.700	1718	215	0.000	ppm	M	C23:0
21	82.054	17155	1132	0.000	ppm	M	C23:0
22	82.564	4252	338	0.000	ppm	M	C23:0

(**d**)

Figure S3. GC-FID chromatograms of (**a**) fatty acid standards and fatty acid profiles in (**b**) MAC-PS, (**c**) SFE-PS, and (**d**) SC-PS extracts.

Table S1. Cell viability responses of RAW 264.7, hTERT fibroblast, HaCaT keratinocyte, DU-145, and HFDPCs to perilla seed extracts at all tested concentrations.

Concentration of perilla seed extracts (mg/mL)	Cell viability (% of control)														
	RAW 264.7			hTERT fibroblasts			HaCaT keratinocytes			DU-145			HFDPCs		
	MAC-PS	SPE-PS	SC-PS	MAC-PS	SPE-PS	SC-PS	MAC-PS	SPE-PS	SC-PS	MAC-PS	SPE-PS	SC-PS	MAC-PS	SPE-PS	SC-PS
**2.000**	0.10 ± 0.03	0.22 ± 0.06	7.16 ± 1.47	2.36 ± 0.09	0.93 ± 0.07	75.99 ± 0.48	0.61 ± 0.05	1.42 ± 0.18	2.50 ± 0.04	16.10 ± 1.01	16.34 ± 2.00	64.90 ± 1.57	35.86 ± 0.67	76.52 ± 1.30	76.49 ± 0.78
**1.000**	0.35 ± 0.05	0.14 ± 0.04	74.88 ± 0.48	46.87 ± 0.45	18.76 ± 0.75	91.57 ± 0.21	2.15 ± 0.03	1.03 ± 0.18	12.48 ± 0.49	27.83 ± 1.83	29.10 ± 0.31	79.70 ± 1.00	37.51 ± 0.77	78.20 ± 1.10	82.10 ± 1.23
**0.500**	0.38 ± 0.05	2.20 ± 0.14	79.90 ± 0.15	59.88 ± 0.12	56.68 ± 1.87	100.16 ± 0.45	4.90 ± 0.08	1.26 ± 0.05	24.25 ± 0.29	38.81 ± 1.27	38.98 ± 1.71	93.13 ± 0.92	46.54 ± 0.37	88.85 ± 1.04	83.84 ± 0.65
**0.250**	61.60 ± 0.13	68.80 ± 1.25	99.04 ± 0.79	91.58 ± 0.17	80.64 ± 0.21	99.06 ± 1.93	40.55 ± 0.80	58.92 ± 0.42	42.32 ± 1.44	74.34 ± 2.27	74.81 ± 1.19	98.02 ± 1.44	77.97 ± 0.54	99.93 ± 0.57	87.21 ± 0.96
**0.125**	81.53 ± 0.33	82.63 ± 0.26	99.94 ± 0.16	92.35 ± 0.18	85.82 ± 0.53	98.94 ± 0.49	81.36 ± 0.19	82.60 ± 0.94	84.32 ± 1.58	88.92 ± 1.63	87.63 ± 2.01	101.56 ± 1.41	91.36 ± 0.19	100.17 ± 0.85	90.56 ± 0.61
**0.063**	87.41 ± 1.57	90.24 ± 0.48	99.66 ± 0.10	91.92 ± 0.20	92.60 ± 1.32	100.01 ± 0.38	85.00 ± 0.05	87.70 ± 1.13	93.38 ± 1.35	94.65 ± 2.16	94.80 ± 1.46	100.63 ± 0.56	98.98 ± 0.66	107.28 ± 0.23	104.80 ± 0.75
**0.031**	95.47 ± 0.35	98.57 ± 0.62	100.03 ± 0.11	93.42 ± 0.18	93.72 ± 0.11	100.24 ± 0.40	92.86 ± 0.21	87.80 ± 0.06	105.00 ± 1.37	97.83 ± 1.21	98.62 ± 0.78	100.53 ± 0.74	107.53 ± 1.02	136.41 ± 1.07	108.11 ± 1.02
**0.016**	95.84 ± 0.26	99.64 ± 0.50	103.70 ± 0.12	94.27 ± 0.24	95.29 ± 0.28	103.44 ± 0.88	93.51 ± 0.86	89.30 ± 0.29	105.71 ± 0.49	104.30 ± 1.10	104.25 ± 2.30	100.45 ± 0.53	101.97 ± 0.69	100.07 ± 0.40	100.97 ± 0.67
**0.008**	102.82 ± 0.34	95.92 ± 0.43	103.40 ± 0.15	92.85 ± 0.11	95.80 ± 0.15	101.66 ± 0.39	93.24 ± 0.21	92.44 ± 0.37	102.83 ± 0.74	98.48 ± 0.74	98.51 ± 0.88	98.14 ± 1.40	100.21 ± 0.31	99.50 ± 0.26	99.27 ± 0.17
**0.004**	101.44 ± 0.41	100.24 ± 0.43	101.32 ± 0.11	93.21 ± 0.22	96.77 ± 0.19	100.28 ± 0.34	95.91 ± 0.33	93.72 ± 0.32	103.05 ± 0.87	98.52 ± 0.93	98.22 ± 0.70	97.98 ± 1.23	101.12 ± 1.23	99.92 ± 0.27	100.52 ± 0.56

Note: Results were expressed as mean ± SD for each sample. Cytotoxicity was evaluated in RAW 264.7, hTERT fibroblasts, HaCaT keratinocytes, DU-145, and HFDPC cells after 24 h of treatment with extract concentrations ranging from 0.004 to 2.000 mg/mL. A concentration of 0.031 mg/mL was selected for proliferation assays due to its non-cytotoxicity and stimulatory effect on HFDPCs. The concentration of 0.125 mg/mL was chosen for experiments, as it maintained cell viability above 80% across all cell types.
